# Epidemiological Trends of Dengue Disease in Brazil (2000–2010): A Systematic Literature Search and Analysis

**DOI:** 10.1371/journal.pntd.0002520

**Published:** 2013-12-19

**Authors:** Maria Glória Teixeira, João Bosco Siqueira,, Germano L. C. Ferreira, Lucia Bricks, Graham Joint

**Affiliations:** 1 Instituto de Saúde Coletiva, Federal University of Bahia, Salvador, Brazil; 2 Universidade Federal de Goiás, Goiânia, Brazil; 3 Sanofi Pasteur, Global Epidemiology, Lyon, France; 4 Sanofi Pasteur, São Paulo, Brazil; 5 Communigen Ltd., The Magdalen Centre, Oxford Science Park, Oxford, United Kingdom; University of South Florida, United States of America

## Abstract

A literature survey and analysis was conducted to describe the epidemiology of dengue disease in Brazil reported between 2000 and 2010. The protocol was registered on PROSPERO (CRD42011001826: http://www.crd.york.ac.uk/prospero/display_record.asp?ID=CRD42011001826). Between 31 July and 4 August 2011, the published literature was searched for epidemiological studies of dengue disease, using specific search strategies for each electronic database. A total of 714 relevant citations were identified, 51 of which fulfilled the inclusion criteria. The epidemiology of dengue disease in Brazil, in this period, was characterized by increases in the geographical spread and incidence of reported cases. The overall increase in dengue disease was accompanied by a rise in the proportion of severe cases. The epidemiological pattern of dengue disease in Brazil is complex and the changes observed during this review period are likely to have been influenced by multiple factors. Several gaps in epidemiological knowledge regarding dengue disease in Brazil were identified that provide avenues for future research, in particular, studies of regional differences, genotype evolution, and age-stratified seroprevalence.

**Systematic Review Registration:**

PROSPERO registration number: CRD42011001826.

## Author Summary

Dengue disease is the most prevalent arthropod-borne viral disease in humans and is a global and national public health concern in Brazil. We conducted this review to consolidate and describe the existing evidence on the epidemiology of dengue disease in Brazil, between 2000 and 2011, to gauge the recent national and regional impact of dengue disease and provide a basis for setting research priorities and prevention efforts. We used well-defined methods to search and identify relevant research, according to predetermined inclusion criteria. Despite control measures, the increased territorial distribution of the mosquito vector and the co-circulation of multiple dengue virus serotypes have resulted in increases in the incidence and distribution of dengue disease. The number of disease-related hospitalizations and deaths has also increased. Efforts to control the increasing disease incidence have been unsuccessful. This review of dengue disease epidemiology will help enhance knowledge and future disease management. Despite the high volume of research retrieved, we have identified several avenues for future research, in particular studies of regional differences, genotype evolution and age-stratified seroprevalence that will improve our knowledge of dengue disease, contribute to a more accurate estimate of global disease incidence, and also inform evidence-based policies for dengue disease prevention.

## Introduction

Dengue disease is an escalating public health problem [1]. Approximately 2·5 billion people live in over 100 endemic countries, predominantly in tropical areas where dengue viruses (DENV) can be transmitted [2]. DENV are arboviruses that are transmitted to humans by infected *Aedes aegypti (Linnaeus)* mosquitoes – the primary vector. Infection with any one of four DENV serotypes (DENV-1, -2, -3, or -4) can produce a spectrum of illness ranging from a mild, non-specific febrile syndrome, to classic dengue fever (DF), or severe disease forms, such as dengue haemorrhagic fever (DHF) and dengue shock syndrome (DSS), that can be fatal. The World Health Organization (WHO) estimates that >50 million dengue infections and >20,000 dengue-related deaths occur annually [1], [3], [4]. A recent disease distribution model has estimated there to be 390 (95% credible interval 284–528) million dengue infections per year, of which 96 million are apparent (i.e., cases manifest any level of clinical or sub-clinical severity) [3]. During 2001–2007, >4 million cases were notified in the Americas, and during 1995–2002, >75% of these cases were reported from Brazil [5], [6].

*Ae. aegypti* was eradicated from Brazil as a result of a Pan American Health Organization (PAHO) programme to control the spread of yellow fever. Additionally, DENV transmission was also suppressed in the Americas during the eradication programme. South American countries became re-infested with *Ae. aegypti* after the programme was discontinued and this, combined with the co-circulation of multiple DENV serotypes, led to the spread of dengue disease across the continent [5], [7]–[9].

In 1982, there was a dengue outbreak in a small city in the northern region of Brazil (Boa Vista/Roraima), which was quickly brought under control and the virus did not spread [10]. In 1986, the re-emergence of DENV-1 in Rio de Janeiro state [11] resulted in over 60,000 reported cases in 1987 and the subsequent spread of DENV increased national public health concerns [12]–[14]. Since the late 1980's the incidence of dengue disease continued to increase; 204,000 cases were reported nationally in 1999 [15], [16]. By 2000, DENV transmission was reported in 22/27 Brazilian states, and the mosquito vector was present in all states [17].

Much of Brazil is affected by a tropical wet and dry climate with high temperatures, high humidity and seasonal variations in rainfall; climate patterns that can provide appropriate conditions for breeding and survival of the *Ae. aegypti* mosquito. The country is divided into five regions (North, Northeast, Central-West, Southeast, and South) comprising 26 states and the federal district containing the capital city, Brasília. In 2000 there were nearly 170 million inhabitants of Brazil, increasing to more than 190 million in 2010 [18], the majority of whom live in the large cities of the Southeast and Northeast regions [19].

The National System for Surveillance and Control of Diseases (SNVS) of Brazil, operates as part of the national health system (Sistema Único de Saúde, or SUS). All reported cases from public health services or private health providers are included in the notification database (Sistema de Informacoes de Agravos de Notificacao [SINAN]), which is openly accessible via the internet [20]. Until 2011, the SNVS adopted the case definitions outlined in WHO guidelines [21], [22]. In 1997, the WHO categorized symptomatic dengue disease as: undifferentiated fever, DF and, DHF [21]. DHF was further classified into four severity grades, with grades III and IV being defined as DSS. However, difficulties in applying the criteria for DHF [23], led the WHO to suggest a new classification based on levels of severity: non-severe dengue disease with or without warning signs, and severe dengue disease [22]. During 2000–2011, both surveillance and hospitalization reporting systems in Brazil used DF and DHF; the surveillance system used an additional classification designated ‘DF with complications’ (DFC) [24]. Importantly, the articles included in this literature analysis that were based on secondary data used these surveillance sources.

Our objectives of this literature search and analysis were to describe the epidemiology of dengue disease (national and regional incidence [by age and sex], seroprevalence and serotype distribution and other relevant epidemiological data) in Brazil during 2000–2011, and to identify gaps in epidemiological knowledge requiring further research.

## Methods

A literature review group, including authors of this contribution, developed a literature survey and analysis protocol based on the preferred reporting items of systematic reviews and meta-analyses (PRISMA) guidelines [25]. Our protocol prescribed well-defined methods to search, identify, and select relevant research, and set predetermined inclusion criteria. The protocol was registered on PROSPERO, an international database of prospectively registered systematic reviews in health and social care managed by the Centre for Reviews and Dissemination, University of York (CRD42011001826: http://www.crd.york.ac.uk/prospero/display_record.asp?ID=CRD42011001826; protocol: http://www.crd.york.ac.uk/PROSPEROFILES/1826_PROTOCOL_20130401.pdf) on 9 December 2011.

### Search strategy and selection criteria

Between 31 July 2011 and 4 August 2011, we searched databases of published literature (Table 1) for epidemiological studies of dengue disease in Brazil. Search strategies for each database were described with reference to the expanded Medical Subject Headings (MeSH) thesaurus, encompassing the terms ‘dengue’, ‘epidemiology’, and ‘Brazil’. Google and Yahoo searches (limited to the first 50 results) were used to identify national and international reports and guidelines, congress abstracts, and grey literature (e.g., Ministry of Health data, lay publications).

**Table 1:** Databases searched for citations relating to dengue disease epidemiology in Brazil.

Database	Website
United States National Library of Medicine and the National Institutes of Health Medical Database	http://www.ncbi.nlm.nih.gov/pubmed/
Excerpta Medica Database (EMBASE)	
MedLine	
Scientific Electronic Library Online (SciELO) – a consolidated electronic publication project that makes available the full text articles from more than 290 scientific journals from Brazil, Chile, Cuba, Spain, Venezuela and other Latin American countries	http://www.scielo.org/php/index.php?lang=en
Virtual Health Library (VHL), an initiative by Brazil-based BIREME (the Latin American and Caribbean Center on Health Sciences Information) that facilitates searches of the Latin American and Caribbean Health Sciences Database (LILACS) and the PAHO Headquarters Library database and other regional health resources	http://regional.bvsalud.org/php/index.php?lang=en
WHO Library database (WHOLIS)	http://dosei.who.int/uhtbin/cgisirsi/3foptRgmQT/7440030/38/1/X/BLASTOFF
Brazilian Ministry of Education: Theses Bank (CAPES)	http://capesdw.capes.gov.br/capesdw/

To reduce selection bias, peer-reviewed contributions in English, Portuguese, or Spanish published between 1 January 2000 and 4 August 2011 were included; no limits by sex, age, ethnicity of study participants, or by study type were imposed. Single-case reports and articles only reporting data prior to 1 January 2000 were excluded. Unpublished reports were included if they were identified in one of the sources listed above. Data from grey materials supplemented that from peer-reviewed literature. Publications not identified in the target databases by the search strategy (e.g., locally published papers) and unpublished data sources meeting the inclusion criteria (e.g., theses, Ministry of Health data) were included if recommended by members of the literature review group. Editorials and data from literature reviews of previously published peer-reviewed studies were excluded.

Duplicates and articles not satisfying the inclusion criteria were removed following review of the titles and abstracts. A further selection was made based on review of the full text from the first selection of references. Included publications were summarised using a data extraction instrument developed as a series of spreadsheets. Due to the expected heterogeneity of eligible studies in terms of selection, and number and classification of cases, a meta-analysis was not conducted. For the purposes of the analysis we defined national epidemics as those years with an incidence/100,000 above the 75th percentile for the period. A trend analysis was conducted on the national incidence and case number data.

## Results and Discussion

**Figure 1:**
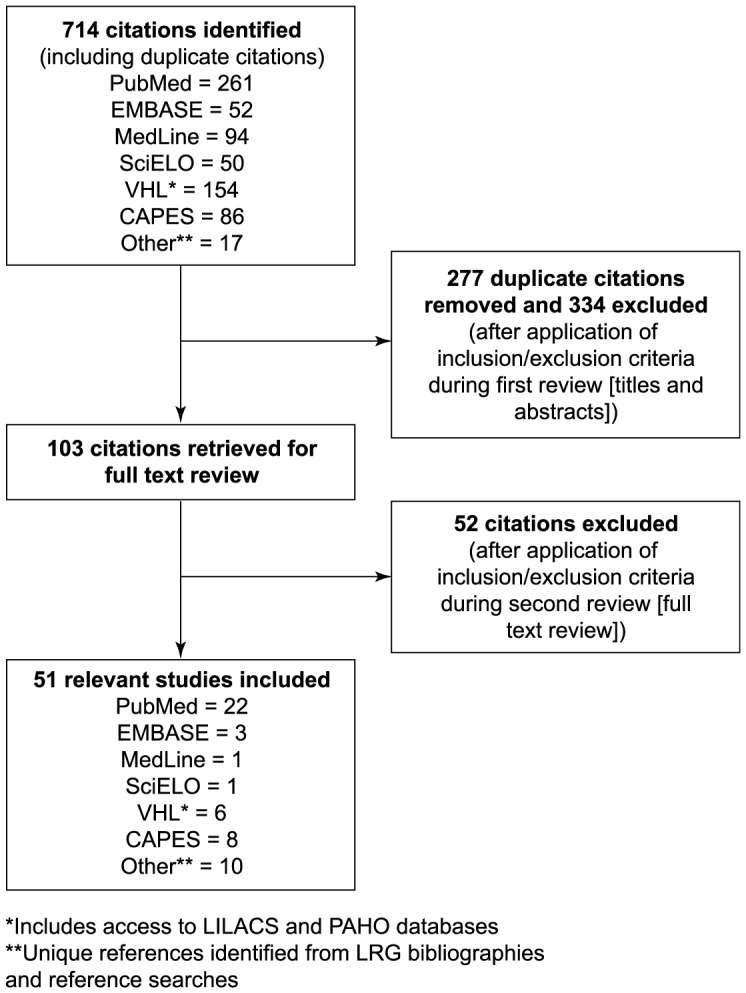
Result of literature search and evaluation of identified studies according to the preferred reporting items of systematic reviews and meta-analyses (PRISMA). All references identified in the on-line database searches were assigned a unique identification number. Following the removal of duplicates and articles that did not satisfy the inclusion criteria from review of the titles and abstracts, the full papers of the first selection of references were retrieved either electronically or in paper form. A further selection was made based on review of the full text of the articles.

We identified 714 relevant citations, 51 of which met the inclusion criteria and were entered into the data extraction instrument (Figure 1; Table S1).

### National epidemiology

**Figure 2:**
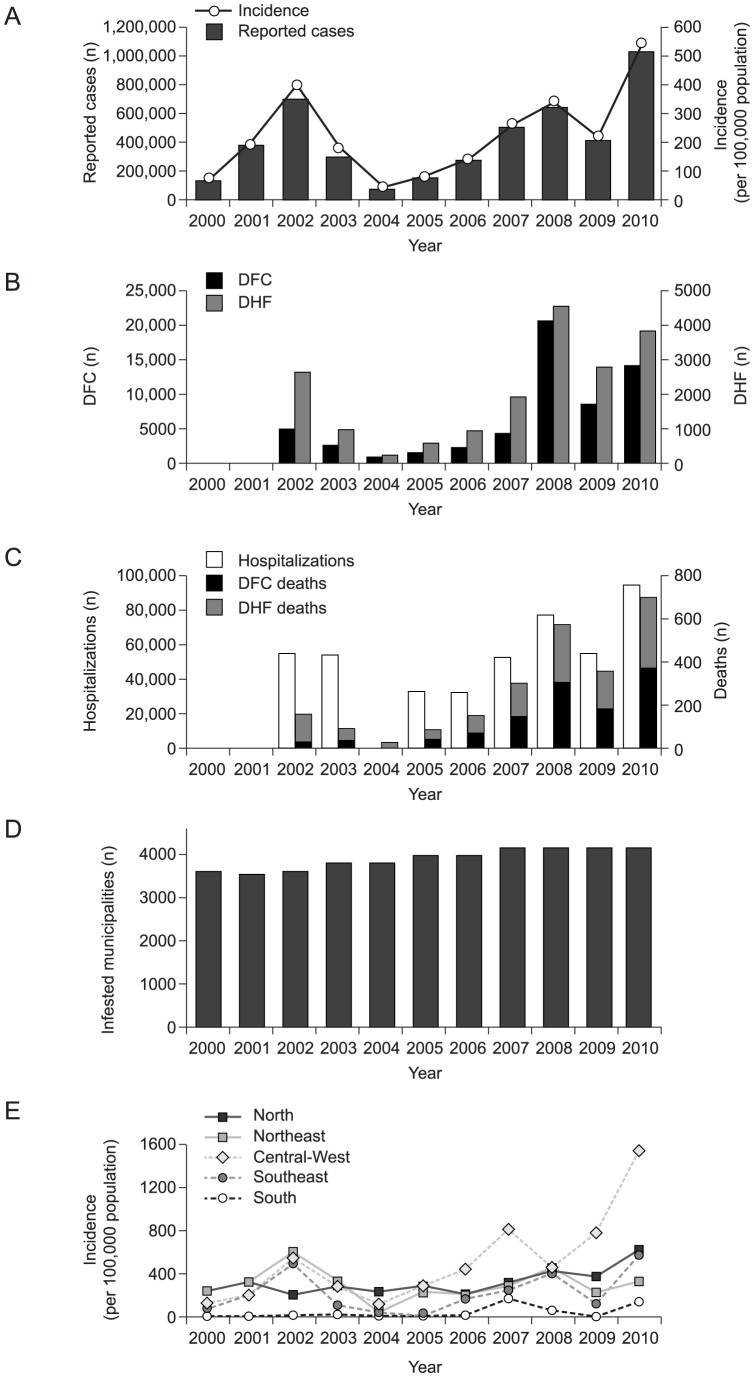
Trends in epidemiology of dengue disease Brazil, 2000–2010. (A) Reported number and average incidence per 100,000 population of probable* cases of dengue disease. (B) Reported number of cases of dengue fever with complications (DFC) and dengue haemorrhagic fever (DHF). (C) Reported number of dengue disease related hospitalizations (DFC+DHF) and deaths due to DFC and DHF. (D) Number of *Ae. aegypti*-infested municipalities. (E). Average incidence of dengue disease per 100,000 population, by region. The epidemiology of dengue disease in Brazil during the review period suggests that incidence and disease severity increased over the decade, although the situation is complicated by national epidemics in 2002, 2008 and 2010. The incidence of dengue disease over the review period reflects the wide distribution of *Ae. aegypti* nationally. In most regions the dengue disease incidence followed national trends. (Adapted from Teixeira 2009 [15] and Siqueira 2010 [26]; additional data supplied by Teixeira MG and Siqueira JB, 2012).

During the period 2000–2010, the incidence of dengue disease in Brazil varied substantially, reaching a peak in 2010 of >1 million cases (538/100,000 inhabitants) and the lowest value was approximately 72,000 cases in 2004 (63.2/100,000 inhabitants) (Table 2, Figure 2A–C, Table S2) [6], [15], [16], [26]–[31]. Despite the yearly variations and cyclical epidemics, trend analysis of the incidence of dengue in Brazil in the period 2000–2010 showed an overall increase in incidence over time that was not statistically significant (β = 12·9/cases per 100,000, p = 0·49). Analysis of the number of cases of dengue disease over the review period shows a growth trend that was not statistically significant (β = 47·984 cases/year, p = 0·25). Nevertheless, the trend analysis suggests a worsening of the problem over time.

**Table 2:** Incidence of dengue disease in Brazil: Summary of national dengue disease incidence data and case numbers and DHF case numbers extracted from source documents.

Year	Parameter	Range	Source of data
2000	Dengue disease (n)	138,388–231,000	6, 16, 27, 31
	Dengue disease (Incidence per 100,000 inhabitants)	92.3–150	15*, 28–30
	DHF (n)	40–888	6, 16, 27, 29
2001	Dengue disease (n)	381,718–413,000	6, 16, 27, 31
	Dengue disease (Incidence per 100,000 inhabitants)	225–254	15*, 28–30
	DHF (n)	630–682	6, 16, 27, 29
2002	Dengue disease (n)	684,527–794,219	6, 16, 26, 27, 31
	Dengue disease (Incidence per 100,000 inhabitants)	335.3–470	15*, 26, 28–30
	DHF (n)	2608–2714	16, 26, 27, 29
2003	Dengue disease (n)	280529–342000	16, 26, 27, 31
	Dengue disease (Incidence per 100,000 inhabitants)	195–200	15*, 29
	DHF (n)	650–913	16, 26, 27, 29
2004	Dengue disease (n)	71,847–113,000	16, 26, 27, 31
	Dengue disease (Incidence per 100,000 inhabitants)	75	15*
	DHF (n)	81–159	16, 26, 27
2005	Dengue disease (n)	134,298–204,000	16, 26, 27, 31
	Dengue disease (Incidence per 100,000 inhabitants)	150	15*
	DHF (n)	463–1395	16, 26, 27
2006	Dengue disease (n)	252725–347000	16, 26, 27
	Dengue disease (Incidence per 100,000 inhabitants)	200	15*
	DHF (n)	642–910	16, 26, 27
2007	Dengue disease (n)	501666–560000	16, 26, 27
	Dengue disease (Incidence per 100,000 inhabitants)	300	15*
	DHF (n)	1541–1907	16, 26, 27
2008	Dengue disease (n)	637,663–806,036	16, 26, 27
	Dengue disease (Incidence per 100,000 inhabitants)	120–336.3	15*, 26
	DHF (n)	647–4502	16, 26, 27
2009	Dengue disease (n)	407,000–411,500	16, 26
	Dengue disease (Incidence per 100,000 population)	205,5–214,9	15*
	DHF (n)	2679	26
2010	Dengue disease (n)	1,027,100	26
	Dengue disease (Incidence per 100,000 inhabitants)	538.4	26
	DHF (n)	3807	26

Empty cells indicate data not reported in source documents.

* Dengue disease incidence data from Teixeira 2009 [15] were estimated from Figure 2. Dengue fever incidence rates (per 100,000 inhabitants) according to geographic regions and year of occurrence. Brazil, 1986–2007.

There were three national epidemics (years with incidence above the 75^th^ percentile for the period [279.95]) in 2002, 2008 and 2010. In 2002 there were 684,527 to 794,219 probable cases of DF, in 2008, 637,663 to 806,036 cases [16], [26], [27], and in 2010 there were over 1 million reported cases (Table 2; Figure 2A) [26]. A trough occurred in 2004 (71,847 to 113,000 cases) [16], [26], [27], [31], representing <10 times the number reported in the peak year, 2010 (Table 2; Figure 2A).

The number of reported severe cases also varied by year and the annual proportion of DF manifest as DHF was 0.1–0.5% over the review period. In 2000, the annual number of DHF cases was between 40 and 4502 [6], [15], [16], [26], [27]. The number of DHF cases during 2000–2010 (>18,000) is striking when compared with data from the previous decade: during the 1990s <1000 cases of DHF were reported [26]. The years in which numbers of DHF cases peaked reflected the national epidemic years for dengue disease described above, with high numbers of DHF cases in 2002 and 2008 (Figure 2B). The 2008 national epidemic of DF/DHF continued with elevated incidence into 2009/2010, with nearly 4000 cases of DHF reported in 2010 [26].

The proportion of severe cases reported is typical of countries in the Americas, but is low compared with Asia where the reported incidence of DHF is much greater [32]. In similar-sized populations, attack rates for severe dengue disease are 18 times greater in Southeast Asia than in the Americas [32]. However, differences in health surveillance system reporting guidelines and variations in case management practices may contribute to the differences in the number of cases reported, and limit the ability to make valid comparisons [33]. In Brazil, DHF cases are defined by strict application of all four criteria from the 1997 WHO guidelines, which is not the case elsewhere [1].

Similarly, hospitalizations related to dengue disease increased over the survey period to >94,000 hospitalizations in 2010 (Figure 2C) [26]. The incidence of dengue-related hospitalization was 31·6/100,000 population during the 2002 national epidemic, approximately 40·8/100,000 during the 2008 national epidemic, and 49·7/100,000 during the 2010 national epidemic [26]. These increases in hospitalization rates during epidemic years might suggest an increase in the severity of dengue disease in Brazil, although an increased awareness during epidemics and a lower threshold for hospitalization might also account for these increases.

The number of dengue-related deaths followed the same patterns as the other epidemiological indices of dengue disease. In 2010, of 13,909 cases classified as DFC and 3807 classified as DHF, there were 370 and 308 fatal cases, respectively. The overall number of DHF- or DFC-related deaths was 678 compared with only 19 in 2004 (Figure 2C) [26].

A seasonal pattern of dengue disease was observed in those studies with available seasonal case distribution data. The highest incidences occurred during January–June [34]–[38], corresponding to the period of highest rainfall and humidity, providing suitable conditions for *Ae. aegypti* breeding and survival. The study by Goncalves Neto et al. [35] showed that 83·3% of dengue disease cases occurred during the rainy season and demonstrated a positive Pearson correlation with the amount of rainfall (r = 0·84) and relative humidity (r = 0·76) and a negative correlation with temperature (r = −0·78).

### Regional epidemiology

We found published regional data for part of the study period from four of the five Brazilian regions [6], [28], [34], [35], [39]–[51]. No published data were recovered for the North region. The available data show that incidence rates varied greatly throughout the country (data not shown; Table S3). In a study of 146 Brazilian cities in October 2006, incidence rates (per 100,000 population) in the 61 cities that reported >500 dengue disease cases ranged between 24·70 (Sao Paulo) and 6222·71 (Campo Grande) [52]. By the end of 2006, 25 of the 27 states had reported local dengue epidemics [15].

The geographic distribution of the *Ae. aegypti* vector has widened over the 11-year review period, involving an increasing number of municipalities (Figure 2D) and this has resulted in a broader regional distribution of dengue disease. In most regions the dengue disease incidence followed national trends (Figure 2E). In the early years of the survey, the Southeast and Northeast regions were most affected by DENV infections, whereas from 2009 more cases were reported from studies within the Central-West region. Incidence rates reported in the South region were consistently lower than in other regions. The distribution of reported cases of dengue disease during the 2010 national epidemic was different from that in the 2002 and 2008 national epidemics with high attack rates observed over larger areas of Brazil [26]. These regional variations in dengue disease incidence are unsurprising given the geographically diverse nature of Brazil with its large variations in climate and population density.

### Demographic patterns of dengue disease in Brazil

**Figure 3:**
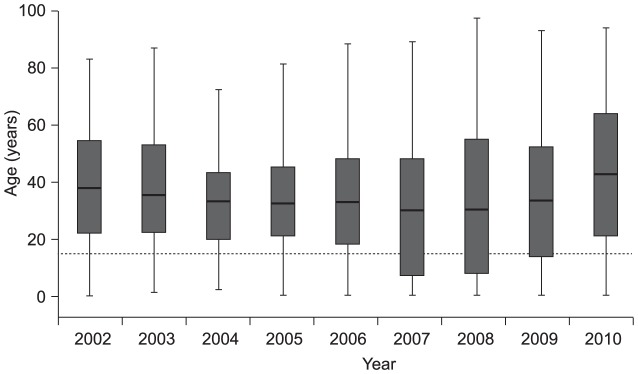
Distribution of reported hospitalized dengue disease cases according to age, Brazil, 2002–2010. A reduction in the first quartile of dengue disease hospitalizations is evident in 2007–2008, although data from 2009 suggest this change may have been transient. Data are median, first and third quartiles, and minimum and maximum ages; the dashed line indicates age 15 years. (Siqueira 2010 [26]. Figure updated and reproduced with kind permission from the Secretariat of Health Surveillance (SVS) of the Ministry of Health of Brazil; additional data supplied by Siqueira JB, 2012).

A change in the age distribution of dengue disease over the survey period was evident from the available data. Young adults were most affected by DF and DHF during 2000–2007 and 2000–2005, respectively (i.e., DHF was coincident with the highest incidence of DF). However, in 2006 the incidence of DHF among children aged <5 years increased (0·47/100,000) and was higher than among those aged 10–19 years and 20–39 years (0·36/100,000 and 0·46/100,000, respectively) [9]. During 1998–2006, most DHF cases were in the 20- to 40-year age group, whereas in 2007 >53% of DHF cases occurred in children <15 years of age [53]. In 2007, a large proportion of cases of dengue-related hospitalizations (40.8%) occurred among those aged <10 years. Furthermore, children aged 5–9 years and 10–14 years showed marked increases in hospitalization rates (68·2 and 60·6/100,000 population, respectively) during the 2008 national epidemic, compared with during the 2002 national epidemic (15·9 and 23·1/100,000 population, respectively) [26]. These hospitalization data are in agreement with the distribution of hospitalizations for dengue disease according to age for 2002–2011 (Figure 3) [26], which suggests a change in age pattern in 2007–2008 (a reduction in the first quartile age) although data from 2009 suggest this change may have been transient. The median age of death from DF was approximately 38 years in 2002 and fell to 30 years between 2007 and 2009 [26].

Regional age-related data from eligible studies are sparse and inter-regional comparisons are difficult (Table 3) [35], [39]–[42], [44], . The most comprehensive data are for 2001–2008 from Ceará state, Northeast region [39]. In 2001, the highest incidence of cases occurred in those aged 20–59 years, whereas in the 2008 national epidemic, those mostly affected were aged <10 years. These data reflect the national changes in age distribution of dengue disease.

**Table 3:** Demographic patterns of incidence of dengue disease: Regional male∶female ratio and age distribution data extracted from source documents.

Year	Location	Region	Male∶ female ratio	Age group (years)					Source of data First author, year^Ref^
				<10	10–19	20–39	40–59	≥60	
1995–2006	State of Pernambuco*	Northeast		11.0%	18.5%	55.0%		15.3%	Cordeiro 2007 [34]
2000	City of Ribeirão Preto	Southeast	0.691						Hino 2010 [47]
2000	City of São Luís	Northeast	1.086						Goncalves Neto 2004 [35]
2000	State of Ceará	Northeast		[65.4]	[129.3]	[263.6†]		[194.8]	Cavalcanti 2011 [39]
2000–2009	City of Vitória	Southeast		7.27%	17.7%	44.1%	24.4%	6.46%	Cardoso 2011 [42]
2000–2009	City of Itabuna	Northeast		44.3%	42.3% [1619.9]	8.00% [1461.7]	4.16%; [1226.1]	1.24% [764.8]	De Souza 2010 [40]
2001	City of Ribeirão Preto	Southeast	0.811						Hino 2010 [47]
2001	City of São Luís	Northeast	0.861						Goncalves Neto 2004 [35]
2001	State of Ceará	Northeast		[174.7]	[321.4]	[659.3†]		[423.4]	Cavalcanti 2011 [39]
2001–2007	City of Anapolis	Central-West		4.0%	13.5%	46.2%	29.0%	7.3%	Santos 2009 [50]
2002	City of Ribeirão Preto	Southeast	0.949						Hino 2010 [47]
2002	City of Recife	Northeast	0.629						Montenegro 2006 [44]
2002	City of São Luís	Northeast	0.819						Goncalves Neto 2004 [35]
2002	State of Ceará	Northeast		[78.5]	[160.6]	[304.3†]		223.3	Cavalcanti 2011 [39]
2003	City of Ribeirão Preto	Southeast	0.893						Hino 2010 [47]
2003	State of Ceará	Northeast		[128]	[250.4]	[416.9†]		[313]	Cavalcanti 2011 [39]
2004	State of Ceará	Northeast		[14.3]	[34.3]	[53.6†]		[39.1]	Cavalcanti 2011 [39]
2005	State of Ceará	Northeast		[126.5]	[198.2]	[365.2†]		[441.5]	Cavalcanti 2011 [39]
2005	City of Goiania‡ §	Central-West		16.9%/4.79%	19.9%/14.4%	36.8%/47.3%	20.2%/24.5%	5.24%/9.0%	Da Silva 2009 [41]
2006	State of Ceará	Northeast		[116]	[247.9]	[412.6†]		[422.2]	Cavalcanti 2011 [39]
2006	City of Goiania‡ §	Central-West		13.1%/8.47%	18.1%/17.7%	36.6%/44.9%	22.0%/20.5%	9.63%/8.47%	Da Silva 2009 [41]
2007	State of Ceará	Northeast		[236.7]	[305.6]	[331.5†]		[249.9]	Cavalcanti 2011 [39]
2007	City of Goiania‡ §	Central-West		6.78%/12.6%	11.9%/16.6%	37.9%/39.4%	33.9%/23.6%	8.5%/7.85%	Da Silva 2009 [41]
2008	State of Ceará	Northeast		[599.4]	[574.4]	[521.9†]		[301]	Cavalcanti 2011 [39]
2008	City of Goiania‡ §	Central-West		6.0%/12.0%	12.7%/15.7%	35.1%/37.1%	31.8%/23.8%	13.3%/11.5%	Da Silva 2009 [41]
2010	City of Santos¶	Southeast	0.5	5.6%	5.6%	38.9%	50%		Romano 2010 [54]

Empty cells indicate data not reported in source documents.

Age group data are given as percentage of total cases and/or [incidence per 100,000 population].

* Age groups are: <10, 10–19, 20–49, and ≥50 years.

† Age group: 20–59 years.

‡ Solidus separates results from two different systems: SINAN (first) and SIH/SUS (second).

§ Data relate to numbers of hospitalizations, as opposed to dengue disease cases.

¶ Age groups are: 0–10, 11–20, 21–40, and >40 years; all cases (n = 18) are virologically confirmed and from one hospital.

Slightly more women than men are affected by dengue disease throughout Brazil [36], which is similar to the sex distribution of reported cases in other Latin American countries [9]. During 2001–2010 the male∶female ratio of reported cases ranged from 0·75–0·82 [9], [26]. Regional data were more variable. In 2000 the ratio was 1·09 in the city of São Luís [35], and 0·5 in the City of Santos in 2010 [54]. Women with dengue disease were slightly older than men (mean age 33·7 years *versus* 30·2 years, respectively; p = 0.019) [37].

### DENV distribution

#### Seroprevalence

Seroprevalence data provide further information to illustrate epidemiological trends (see *Socio-demographic factors* below). Population seroprevalence estimates varied throughout Brazil during the decade analysed. In individuals aged 18–65 years, the highest seroprevalence rates were reported in the cities of Mossoró and Caruaru (97·8% and 94·5%, respectively) with lower seroprevalence reported in Rio Branco (69·2%) and Macapá (48·4%) [55]. In serological surveys of volunteers without DF symptoms in Goiânia, seroprevalence was 29·5% in 2001 and 37·3% in 2002 [56]. In Recife, a large urban centre, during 2004–2006, 354 (53·8%) of 658 patients with suspected DENV infections had antibodies to DENV, of which 175 (49·4%) were characterized as primary infections and 179 (50·6%) as secondary infections [36]. In 2002, the seroprevalence in Recife was 76·3% (45 cases) [44]. Few age-specific seroprevalence data were reported in studies included in our analysis.

Seroprevalence data also reveal that dengue disease is under-reported. Current passive surveillance systems do not report on mildly symptomatic and non-specific febrile cases and do not represent the true rate of infection and transmission. Based on the findings of a seroepidemiological study in Recife conducted between August and September 2006, Rodriguez-Barraquer *et al.* calculated that <10% of infections may be reported [13]. Comparing the estimated number of individuals with DENV antibodies in three districts of Belo Horizonte in 1996–2006 (79,000) with the number of reported cases (32,330), Pessanha *et al.* suggested that the number of seropositive cases is 2·5 times higher than the number of reported cases [57].

#### Serotype distribution

**Figure 4:**
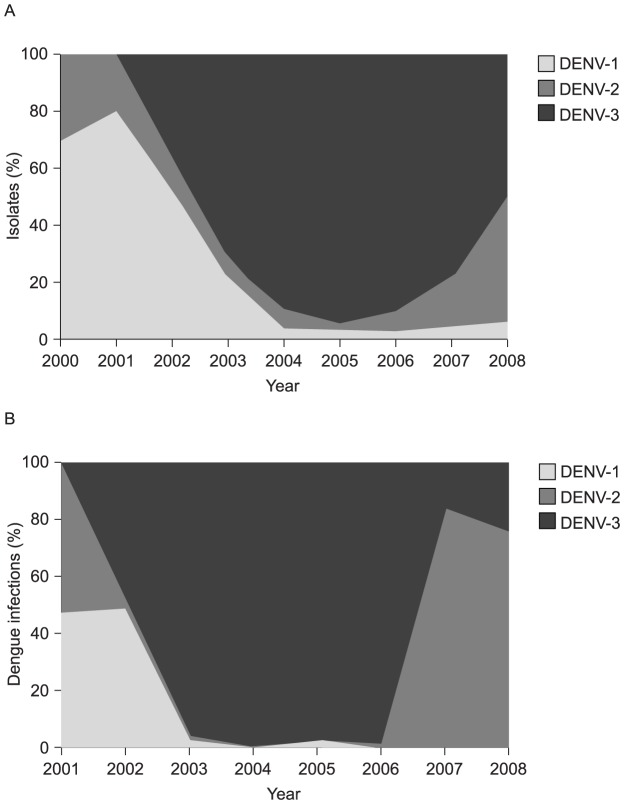
Change in pattern of circulating dengue virus (DENV) serotypes by year, (A) Brazil, 2000–2008 and (B) the Northeast region state of Ceará, 2001–2008. The Brazilian Ministry of Health data from 2000–2008, indicated co-circulation of multiple DENV serotypes with a high proportion of DENV-1 incidence at the beginning of the decade; DENV-3 became predominant from 2003 and DENV-2 was important from 2007. DENV-4 was not present in Brazil until 2011. A similar pattern was observed in the Northeast region state of Ceará. (A: data supplied by Teixeira MG, 2012; B: Cavalcanti 2011 [39]).

National serotype incidence data were not reported in publications identified by the review protocol. Until 2008, most dengue disease diagnoses in Brazil were made using clinical and epidemiological criteria, as isolation and identification of DENV by polymerase chain reaction was scarce. The Brazilian Ministry of Health compiled a series of DENV isolations from 2000–2008, indicating a high proportion of DENV-1 incidence at the beginning of the decade; DENV-3 became predominant from 2003 and DENV-2 was important from 2007 (Figure 4A). The studies we reviewed also indicate a shift to DENV-3 predominance towards the middle of the decade across Brazil, with DENV-2 becoming more important in later outbreaks.

Serotype distribution data reveal trends similar to the national trends, with some local differences (Table 4) [6], [34], [36], [37], [39], [45], [49], [58]–[61]. The most comprehensive regional data are available for the Northeast region states of Ceará [39] and Pernambuco [34], and the Central-West region city of Goiânia [45]. In Ceará a similar pattern of serotype change was reported during 2000–2003, with a shift from DENV-1 and -2 to DENV-3 (Figure 4B). By 2003, almost all infections in the Northeast region were caused by DENV-3, as were three-quarters of those in Goiânia. In contrast, in Rio de Janeiro (Southeast region), the serotype shift may have occurred earlier, with DENV-3 accounting for 99% of infections in 2001 [59] and 2002 [6]. However, for the period 2001–2002, Passos *et al.* reported only 65·7% DENV-3 serotypes for Rio de Janiero [60]. In the North region city of Manaus, Amazonas state, an outbreak in 2006–2007 was attributed to DENV-3, comprising 100% of the serotypes identified in 2006, falling to 78·7% in 2007 [61]. Data from Ceará state are also available for the second half of the survey period (to 2008) [39]. These show a shift from DENV-3 to DENV-2 in 2007 and 2008. Regional data for 2007 onwards, other than those for Ceará state, were not published during the survey period.

**Table 4:** Regional DENV serotype distribution.

Year	Location	Region	DENV-1 (%)	DENV-2 (%)	DENV-3 (%)	Source of data First author, year[Ref]
2000	State of Pernambuco	Northeast	72	28		Cordeiro 2007 [34]
2000	City of Goiânia	Central-West	78.3	21.7		Feres 2006 [45]
2000	Municipality of Belo Horizonte	Southeast	76.9 (1 or 2)			Cunha 2008: calculated [58]
			19.7	3.4		
2000–2001	State of Rio de Janeiro	Southeast	62.7	24.3	13	De Simone 2004 [37]
2000–2002	State of Piaui	Northeast	64	31	5	De Castro 2003 [49]
2001	State of Pernambuco	Northeast	76	24		Cordeiro 2007 [34]
2001	State of Ceará	Northeast	47.7	52.3	0	Cavalcanti 2011 [39]
2001	City of Goiânia	Central-West	78.8	21.2		Feres 2006 [45]
2001	Rio de Janeiro	Southeast	0.60	0.30	99	Nogueira 2005 [59]
2001–2002	Rio de Janeiro	Southeast	17.1	17.1	65.7	Passos 2004 [60]
2002	State of Pernambuco	Northeast	18	9	73	Cordeiro 2007 [34]
2002	State of Ceará	Northeast	48.5	7.4	44.1	Cavalcanti 2011 [39]
2002	City of Goiânia	Central-West	90.7	6.6	2.7	Feres 2006 [45]
2002	Rio de Janeiro	Southeast	0.93	0.31	98.8	Nogueira 2002 [6]
2003	State of Pernambuco	Northeast	1	1	98	Cordeiro 2007 [34]
2003	State of Ceará	Northeast	1.9	1.9	96.2	Cavalcanti 2011 [39]
2003	City of Goiânia	Central-West	17.4	5.8	76.8	Feres 2006 [45]
2004	State of Pernambuco	Northeast	0	0	100	Cordeiro 2007 [34]
2004	State of Ceará	Northeast	0	0	100	Cavalcanti 2011 [39]
2004–2006	Recife, Pernambuco	Northeast	0	0	100	Cordeiro 2007 [36]
2005	State of Pernambuco	Northeast	5	0	95	Cordeiro 2007 [34]
2005	State of Ceará	Northeast	2.5	0	97.5	Cavalcanti 2011 [39]
2006	State of Pernambuco	Northeast	0	6	94	Cordeiro 2007 [34]
2006	State of Ceará	Northeast	0	1.4	98.6	Cavalcanti 2011 [39]
2006	City of Manaus	North	0	0	100	Rocha 2009 [61]
2007	State of Ceará	Northeast	0	84	16	Cavalcanti 2011 [39]
2007	City of Manaus	North	8.5	12.8	78.7	Rocha 2009 [61]
2008	State of Ceará	Northeast	0	76.1	23.9	Cavalcanti 2011 [39]

Regional data extracted from source documents for distribution of DENV-1, 2 and 3 serotypes. DENV-4 was not present in Brazil until 2011.

DENV, dengue viruses.

A report of the first DENV-4 isolate for 25 years in Amazonas in 2008 [62], was followed in July 2010 by its re-emergence in Boa Vista, the capital of Roraima State, after an absence of 28 years [63]. DENV-4 infections have since been reported in the Northeast (Piauí, Pernambuco, Bahia, and Ceará) and the Southeast (Rio de Janeiro and São Paulo) [64]. A serotype-specific NS1 enzyme-linked immunosorbent assay test has been introduced in some states by the Brazilian Ministry of Health as a screening tool to aid determination of the circulating serotypes.

An increase in the magnitude of national epidemics and in the severity of dengue disease in Brazil was observed during the review period (Figure 2A–F). It has been suggested that severe forms of dengue disease in children may be linked to an increased prevalence of DENV-2 versus DENV-3 [53]. However, we do not believe that changes in circulating DENV serotypes are solely responsible for the changes in incidence of DHF observed during this review period. The changes observed during this review period are likely to have been influenced by multiple factors, including regional variations in circulating DENV serotypes, virulence of viral strains, serotype-specific herd immunity in different age groups, and the density of the vector population.

Several studies reported clinical differences in patients with dengue disease associated with distinct DENV serotypes. Pereira *et al.* reported that individuals infected by DENV-3 presented with signs of more severe disease than those associated with DENV-1 or DENV-2 [65]. However, a study by Feres *et al.* in all age groups (age range, 1–60 years) diagnosed with dengue disease in a region of central Brazil, found that the emergence of DENV-3 in this region was not associated with increased disease severity [45].

Although an increase in the severity of dengue disease outcomes in patients with a secondary infection due to a different serotype has been proposed [66], secondary infection was not a predictor of severity in a cohort of adults with confirmed dengue disease (predominantly infected with DENV-3) in central Brazil in 2005 [67]. The relationship between primary and secondary infection, the infecting DENV serotype, and disease severity remains unclear.

Few age-specific serotype data were reported in published studies. In the Greater Metropolitan Region of the State of Rio de Janeiro in 2000–2001, 5324 serum samples were analysed from patients with suspected dengue disease [37]. The mean ages of patients according to infecting serotype were not significantly different (p = 0.108): DENV-1 (30·9±15·9 years), DENV-2 (34·3±15·0 years), and DENV-3 (30·9±14·6 years).

#### Socio-demographic factors

Several studies examined associations between the risk for dengue disease and socio-economic, demographic and infrastructure characteristics. A matched case–control study conducted in Salvador (2002–2003) and Fortaleza (2003–2005) in DENV seropositive individuals demonstrated a significant association between DHF and both high income and increased years of schooling [68]. In another study one-storey homes and a high number of residents per household were identified risk factors for dengue disease [69]. However, Mondini *et al.* found that DENV transmission was independent of socio-economic strata for the years within the survey period [70]. In a study of DENV-3 emergence and dispersion dynamics in the state of Bahia, viral circulation intensity was strongly dependent on increased population density and availability of susceptible individuals [71]. Teixeira *et al.* demonstrated a high risk for dengue disease in towns characterized by urbanization, poor sewer networks, and limited piped water supplies [72].

In Belo Horizonte, 89,607 cases registered in the surveillance system from 1996–2002 were analysed according to defined high- and low-risk areas [73]. Factors significantly associated with high-risk compared with low-risk areas were lower income of the head of the family, higher household density, and larger proportion of children and elderly women [73]. A seroepidemiological study of a random sample of 627 individuals during January 2000 in the same municipality, showed that low income was also associated with high seroprevalence rates. Other variables associated with high seroprevalence rates were residence in horizontal residential buildings with vector infestation and a lack of spatial mobility of residents [58]. During 2005–2006, a household survey was performed in 2833 individuals aged 5–64 years in three diverse socio-economic and environmental areas of Recife. The DENV seroprevalence was 91·1%, 87·4%, and 74·3% in the deprived, intermediate, and high socio-economic areas, respectively, revealing an inverse relationship between high seroprevalence and low socio-economic status [74]. In a similar serological survey in Recife conducted between August and September 2006, three neighbourhoods were selected to represent low (area 1), medium (area 2), and high (area 3) socio-economic areas. Among the 1427 individuals included (aged 5–20 years), seroprevalence was 85%, 70%, and 82% in areas 1, 2, and 3, respectively [12]. In a study in three health districts in the city of Belo Horizonte conducted among 709 individuals between June 2006 and March 2007, seroprevalence was 11·9% (95% confidence interval 9·7–14·6). Seropositivity was associated with construction type (apartment or house/shanty; apartment was a protection factor) and with an elevated health vulnerability index for the location of the dwelling, but was not associated with sex, age, or family income [57].

Our literature survey and analysis reveals heterogeneity in the incidence of dengue disease over time and space that is indicative of the complexity of risk factors involved in disease transmission. However, it is likely that unplanned urbanization and changes in land use (deforestation) play a significant role in raising the incidence and prevalence of dengue disease [72].

Only two of the studies selected for analysis examined the relationship between ethnicity and susceptibility to dengue disease. One study found that both self-defined Afro-Brazilian ethnicity and African ancestry were protective for DHF after controlling for income level [75]. A second study showed that the risk of DHF was 4.6 times higher in those of white ethnicity than those of Afro-Brazilian/African ethnicity [68].

With regard to the risk associated with comorbidities, an association between diabetes, allergy treated with steroids, and hypertension (in those with Afro-Brazilian/African ancestry) and an increased risk for DHF was demonstrated in a matched case–control study conducted in Salvador (2002–2003) and Fortaleza (2003–2005) in individuals with a serologically confirmed history of dengue disease [68].

#### Effectiveness of vector-control measures

After detection of DENV-3, in Rio de Janeiro in 2000, and the co-circulation of three serotypes (DENV-1, DENV-2, and DENV-3), the Ministry of Health established the National Dengue Control Programme (PNCD) in 2002 to implement new strategies and intensify existing plans with greater operational scope [76]. Pessanha *et al.* found a reduction in the number of municipalities with dengue incidence >100/100,000 inhabitants from 66·10% in 2001–2002 (before PNCD implementation) to 48·97% in 2003–2006 (after implementation) [77].

### Strengths and limitations of this survey and analysis

Despite some gaps, our literature survey and analysis provides a comprehensive overview of the evolving epidemiology of dengue disease in Brazil over the period 2000–2011. This study has several important strengths. Our survey was thorough; we screened >700 articles to identify relevant publications and we developed a comprehensive data extraction instrument to facilitate the capture of all relevant data.

Nevertheless, the lack of comprehensive and continuous data for the survey period limits our ability to make comparisons and draw firm conclusions over the years, across regions, and among different ages. For example, age-stratified data were not reported systematically and age range boundaries differed by study. Therefore, although we can suggest trends in age distribution, it is not possible to directly compare data from the selected publications.

The inclusion of publications in three languages reduced selection bias in our literature review and analysis. However, despite the inclusion of PhD dissertations and theses there is a bias towards published articles. An assessment of quality of evidence was not carried out and potential weaknesses of some studies such as inadequately described case selection, small sample sizes, and unspecified statistical methods were not reasons for exclusion. Consequently, any limitations of the original studies are carried forward into our review.

Many of the studies relied on data reported by passive surveillance systems, which can vary between regions and over time [33] and may misrepresent the number of cases due to changes in reporting behaviour and misclassifications.

### Avenues for future research

Our literature survey and analysis identified several knowledge gaps, which indicate potential avenues for future study. In particular, there are gaps relating to the regional incidence of dengue disease in Brazil, national and regional age-related data, and national and regional serotype information. Further epidemiological studies may help to clarify and define regional differences.

The large increase in the number of DHF cases and the shift in age distribution of DHF towards younger age groups that occurred during the 2007–2008 national epidemic warrant explanation. One possibility is that the change in circulating DENV serotypes over time may have affected the pattern of dengue disease epidemiology in Brazil [78]. Age-stratified seroprevalence studies will improve assessment of the level of transmission and inapparent infection, as well as providing information relating to the age shift.

Further studies into the risk factors for dengue disease and its severity are also important. For example, in Southeast Asia, DENV infection has been more widespread for a longer period of time than in the Americas, creating a large group of individuals likely to experience a second or third infection [32]. These secondary infections carry an increased risk of severe dengue disease. The data in this review do not address the Southeast Asian experience and further examination as to whether this phenomenon is replicated in Brazil is required. In addition, few studies in the review specifically measured the effects of urbanization in Brazil, with effects only inferred from studies of other socio-demographic factors. The diversity of ethnic backgrounds within the population suggests that further genetic studies are warranted to determine whether ethnicity affects the clinical expression of dengue disease and the risk for severe outcomes. Studies are also required to clearly define associations with other diseases if comorbidity screening is to be used to identify patients at a greater risk of developing DHF.

We acknowledge that there are gaps in our epidemiological knowledge of dengue disease in Brazil, due, in part (as in many other countries) to the inherent weaknesses of passive surveillance systems. The majority of infections are clinically non-specific consequently dengue disease is often mis-diagnosed during inter-epidemic periods [8]. The findings presented here are in broad agreement with those of Honório *et al.* [79], who found only 23·3% of infections were symptomatic, and with Lima *et al.* [80], who showed that the number of cases reported for the Southeast region of Brazil under-represented the number of infected individuals. This was also found in studies conducted in other countries [81]. Only when an epidemic occurs is the full spectrum of the disease recognised. Consequently, the disease is likely to be under-reported during inter-epidemic periods but over-reported during epidemics [82]. Overall, we believe the national surveillance data under-estimate the true incidence of DENV infections. However, extensive representative serological surveys are required to estimate the true rate of infection and transmission and, thus, despite its drawbacks, passive reporting is important for the identification of disease trends over time.

### Conclusions

Our review and analysis of the epidemiology of dengue disease in Brazil during the past decade suggests an overall increase in the distribution and severity of dengue disease. During the last decade (2000–2010), a total number of 8,440,253 cases were reported (the highest figure in the history of dengue disease in this region) with the highest number of severe cases (221,043; 2.6%) and fatal cases (3058; 0.036% of the total reported cases and 1.38% of the severe cases) [83]. The 1588 cases of severe dengue disease and 163 deaths reported as of epidemiological week 8 in 2011, represent 67% and 73%, respectively, of the total cases registered in the Americas [84]. The co-circulation of multiple DENV serotypes and high dengue disease endemicity may be responsible for the increased occurrence of severe forms of dengue disease and increases in the numbers of dengue disease-related hospitalizations. In addition, the increase in the number of severe cases of dengue disease and a shift in age group predominance of severe forms observed during 2007/08 confirm that dengue disease must remain a public health priority in Brazil.

Even though the studies included in this literature review have improved our understanding of the epidemiology of dengue disease in Brazil, further studies are required to clarify the epidemiological pattern and to understand regional epidemiological differences, the diversity of genotypes of circulating serotypes and the extent of herd immunity by age group. Our review has highlighted the main epidemiological characteristics of dengue in Brazil in the first decade of this century and revealed that the epidemiological pattern of dengue disease in Brazil is complex. The changes observed are likely to have been the result of multiple factors, which still require elucidation.

## Supporting Information

Checklist S1 PRISMA 2009 checklist.
(PDF)

Table S1 Citations used in the literature analysis.
(PDF)

Table S2 Incidence of dengue disease in Brazil: national data.
(PDF)

Table S3 Incidence of dengue disease in Brazil: regional data.
(PDF)
