# Supplementary material for: Epidemiological Trends of Dengue Disease in Brazil (2000–2010): A Systematic Literature Search and Analysis
Source: PLoS Negl Trop Dis. 2013 Dec 19;7(12):e2520. doi: 10.1371/journal.pntd.0002520 (PMC3871634; doi:10.1371/journal.pntd.0002520)
Supplement: Table S1 — Citations used in the literature analysis. (PDF) [file pntd.0002520.s002.pdf]

Supplementary Table S1. Citations used in the literature analysis.

Citation	Reference No.
Blanton RE, Silva LK, Morato VG, Parrado AR, Dias JP, et al. (2008) Genetic ancestry and income are associated with dengue hemorrhagic fever in a highly admixed population. Eur J Hum Genet 16: 762–765. doi: http://dx.doi.org/10.1038/ejhg.2008.4.	75
Braga C, Luna CF, Martelli CM, de Souza WV, Cordeiro MT, et al. (2010) Seroprevalence and risk factors for dengue infection in socio-economically distinct areas of Recife, Brazil. Acta Trop 113: 234–240. doi: http://dx.doi.org/10.1016/j.actatropica.2009.10.021.	74
Cardoso IM, Cabidelle AD, Borges PD, Lang CF, Calenti FG, et al. (2011) Dengue: clinical forms and risk groups in a high incidence city in the southeastern region of Brazil. Rev Soc Bras Med Trop 44: 430–435. doi: http://dx.doi.org/10.1590/S0037-86822011005000044.	42
Cavalcanti LP, Vilar D, Souza-Santos R, Teixeira MG. (2011) Change in age pattern of persons with dengue, northeastern Brazil. Emerg Infect Dis 17: 132–134. doi: http://dx.doi.org/10.3201/eid1701.100321.	39
Coelho GE, Burattini MN, Teixeira MG, Coutinho FA, Massad E. (2008) Dynamics of the 2006/2007 dengue outbreak in Brazil. Mem Inst Oswaldo Cruz; 103: 535–539. doi: http://dx.doi.org/10.1590/S0074-02762008000600004.	52
Cordeiro MT, Schatzmayr HG, Nogueira RM, Oliveira VF, Melo WT, et al. (2007) Dengue and dengue hemorrhagic fever in the State of Pernambuco, 1995–2006. Rev Soc Bras Med Trop 40: 605–611. doi: http://dx.doi.org/10.1371/journal.pntd.0000890.	34
Cordeiro MT, Silva AM, Brito CA, Nascimento EJ, Magalhães MC et al. (2007) Characterization of a dengue patient cohort in Recife, Brazil. Am J Trop Med Hyg 77: 1128–1134. Available: http://www.ajtmh.org/content/77/6/1128.long. Accessed: 30 September 2013.	36
Cunha MdCM, Caiaffa WT, Oliveira CdL, Kroon EG, Pessanha JEM, et al. (2008) [Associated factors to infection by dengue virus in the municipality of Belo Horizonte, State of Minas Gerais, Brazil: individual characteristics and intra-urban differences]. Epidemiol Serv Saúde 17: 217–230 [Portuguese].	58
Da Fonseca GF. (2009) Dengue fever in Brazil: trends, surveillance and outbreak of 2008 [thesis]. Goiânia, Brazil: Universidade Federal de Goiás 1 v. 61 p.	27
Da Silva JB. (2009) Quality of information systems and SINAN-SIH SUS and the proportion of severe cases of dengue in the city of Goiânia-Go, 2005–2008: estimation by the method of capture–recapture [thesis]. Goiânia, Brazil: Universidade Federal de Goiá 1 v. 56 p.	41
de Castro JA, de Andrade HM, do Monte SJ, da Silva AS, Gomes KC, et al. (2003) Dengue viruses activity in Piaui, Brazil. Mem Inst Oswaldo Cruz 98: 1021–1023. doi: http://dx.doi.org/10.1590/S0074-02762003000800007.	49
de Mattos Almeida MC, Caiaffa WT, Assuncao RM, Proietti FA. (2007) Spatial vulnerability to dengue in a Brazilian urban area during a 7-year surveillance. J Urban Health 84: 334–345. doi: http://dx.doi.org/10.1007/s11524-006-9154-2.	73
de Melo PR, Reis EA, Ciuffo IA, Goes M, Blanton RE, et al. (2007) The dynamics of dengue virus serotype 3 introduction and dispersion in the state of Bahia, Brazil. Mem Inst Oswaldo Cruz 102: 905–912. doi: http://dx.doi.org/10.1590/S0074-02762007000800003	71
De Simone TS, Nogueira RM, Araújo ES, Guimarães FR, Santos FB, et al. (2004) Dengue virus surveillance: the co-circulation of DENV-1, DENV-2 and DENV-3 in the State of Rio de Janeiro, Brazil. Trans R Soc Trop Med Hyg 98: 553–562. doi: http://dx.doi.org/10.1016/j.trstmh.2003.09.003.	37

De Souza TsBB, Dias JP. (2010) [Epidemic profile of dengue in the municipal district of Itabuna (BA), 2000 Jun. 2009]. Rev Baianade Saude Publica 34:665–681 [Portuguese].	40
Dias JP. (2006) Evaluation of the effectiveness of the eradication of Aedes Aegypti, programme Brazil, 1996–2002 [thesis]. Salvador, Brazil: Universidade Federal da Bahia 1 v. 79 p.	55
dos Santos SL. (2003) Assessment of dengue control actions: critical aspects and perception of population – a case study in a northeastern town [thesis]. Fiocruz, Brazil: Magellan Aggeu Research Center 1 v. 132 p.	28
Feres VC, Martelli CM, Turchi MD, Junior JB, Nogueira RM, et al. (2006) Laboratory surveillance of dengue virus in Central Brazil, 1994–2003. J Clin Virol 37: 179–183. doi: http://dx.doi.org/10.1016/j.jcv.2006.07.004.	45
Figueiredo LTM. (2010) Dengue in Brazil during 1999–2009: A review. Dengue Bulletin 34: 6–12. Available: http://repository.searo.who.int/handle/123456789/16161. Accessed: 30 September 2013.	16
Figueiredo MA, Rodrigues LC, Barreto ML, Lima JW, Costa MC, et al. (2010) Allergies and diabetes as risk factors for dengue hemorrhagic fever: results of a case control study. PLoS Negl Trop Dis 4: e699. doi: http://dx.doi.org/10.1371/journal.pntd.0000699.	68
Figueiredo RM, Naveca FG, Bastos MS, Melo MN, Viana SS, et al. (2008) Dengue virus type 4, Manaus, Brazil. Emerg Infect Dis 14: 667–669. doi: http://dx.doi.org/10.3201/eid1404.071185.	62
Gasparetti MV, Castro R, Castro SA, Maria GS, Cotait AdAL, et al. (2007) Dengue incidence in ABCD region. Arq Med ABC 32: 70–73.	46
Gonçalves Neto VS, Rebêlo JM. (2004) [Epidemiological characteristics of dengue in the Municipality of São Luis, Maranhao, Brazil, 1997–2002]. Cad Saude Publica 20: 1424–1431 [Portuguese]. doi: http://dx.doi.org/10.1590/S0102-311X2004000500039.	35
Guilarde AO, Turchi MD, Siqueira JB, Jr, Feres VC, Rocha B, et al. (2008) Dengue and dengue hemorrhagic fever among adults: clinical outcomes related to viremia, serotypes, and antibody response. J Infect Dis 197: 817–824. doi: http://dx.doi.org/10.1086/528805.	67
Hino P, dos Santos CC, dos Santos MO, da Cunha TN, dos Santos CB. (2010) [Temporal evolution of dengue fever in Ribeirao Preto, Sao Paulo State, 1994–2003]. Cien Saude Colet 15: 233–238 [Portuguese]. doi: http://dx.doi.org/10.1590/S1413-81232010000100028.	47
Koyama AM, Baldissera VDA. (2009) [Description of the cases of dengue fever in the southern states of Brazil, 2001–2005]. Arquivos de Ciências da Saúde da UNIPAR 13:125–132 [Portuguese].	43
Mondini A, Chiaravalloti NF. (2007) [Socioeconomic variables and dengue transmission]. Rev Saude Publica 41: 923–930 [Portuguese]. doi: http://dx.doi.org/10.1590/S0034-89102007000600006.	70
Mondini A, Chiaravalloti-Neto F. (2008) Spatial correlation of incidence of dengue with socioeconomic, demographic and environmental variables in a Brazilian city. Sci Total Environ 393(2–3): 241–248. doi: http://dx.doi.org/10.1016/j.scitotenv.2008.01.010.	69
Monteiro ESC, Coelho MnE, Cunha ISd, Cavalcante MdAS, Carvalho FAcdA. (2009) [Epidemiological and vector-related indicators of dengue fever in Teresina city, Piaui State, Brazil, from 2002 to 2006]. Epidemiol Serv Saúde 18: 365–374 [Portuguese].	51
Montenegro D, Lacerda HR, Lira TM, Oliveira DS, Lima AA, et al. (2006) [Clinical and epidemiological aspects of the dengue epidemic in Recife, PE, 2002]. Rev Soc Bras Med Trop 39: 9–13 [Portuguese]. doi: http://dx.doi.org/10.1590/S0037-86822006000100002.	44

Moraes GH. (2009) Death by severe dengue in Brazil, 2000 to 2005: Correlation of notification in two information systems and case-control study of factors associated with [thesis]. Seattle, WA, USA: University of Washington 1 v. 129 p.	31
Nogueira RM, Schatzmayr HG, de Filippis AM, Dos Santos FB, da Cunha RV, et al. (2005) Dengue virus type 3, Brazil, 2002. Emerg Infect Dis 11: 1376–1381. doi: http://dx.doi.org/10.3201/eid1109.041043.	59
Nogueira RMR, Miagostovich MP, Schatzmayr HG. (2002) Dengue viruses in Brazil. Dengue Bulletin 26: 77–83. Available: http://repository.searo.who.int/handle/123456789/15884. Accessed 30 September 2013.	6
Pan American Health Organization. (2011) Epidemiological alert: Update on dengue in the Americas. 19 August 2011. Available: http://new.paho.org/hq/index.php?option=com_docman&task=doc_view&gid=14579&Itemid=1091. Accessed: 27 September 2013.	84
Passos MN, Santos LM, Pereira MR, Casali CG, Fortes BP, Ortiz Valencia LI, et al. (2004) [Clinical differences observed in patients with dengue caused by different serotypes in the epidemic of 2001/2002, occurred in Rio de Janeiro]. Rev Soc Bras Med Trop 37: 293–295 [Portuguese]. doi: http://dx.doi.org/10.1590/S0037-86822004000400001.	60
Pessanha JE, Caiaffa WT, Cesar CC, Proietti FA. (2009) [Evaluation of the Brazilian National Dengue Control Plan]. Cad Saude Publica 25: 1637–1641 [Portuguese]. doi: http://dx.doi.org/10.1590/S0102-311X2009000700024.	77
Pessanha JE, Caiaffa WT, Kroon EG, Proietti FA. (2010) [Dengue fever in three sanitary districts in the city of Belo Horizonte, Brazil: a population-based seroepidemiological survey, 2006 to 2007]. Rev Panam Salud Publica 27: 252–258 [Portuguese]. doi: http://dx.doi.org/10.1590/S1020-49892010000400003	57
Rocha LA, Tauil PL. (2009) [Dengue in children: clinical and epidemiological characteristics, Manaus, State of Amazonas, 2006 and 2007]. Rev Soc Bras Med Trop 42: 18–22 [Portuguese]. doi: http://dx.doi.org/10.1590/S0037-86822004000400001.	61
Rodriguez-Barraquer I, Cordeiro MT, Braga C, de Souza WV, Marques ET, et al. (2011) From re-emergence to hyperendemicity: the natural history of the dengue epidemic in Brazil. PLoS Negl Trop Dis 5: e935. doi: http://dx.doi.org/10.1371/journal.pntd.0000935.	13
Romano CM, de Matos AM, Araujo ES, Villas-Boas LS, da Silva WC, et al. (2010) Characterization of Dengue virus type 2: new insights on the 2010 Brazilian epidemic. PLoS One 5: e11811. doi: http://dx.doi.org/10.1371/journal.pone.0011811.	54
Sampaio AS. (2008) [The epidemiologic situation of dengue in Salvador (BA), 2000–2005]. Rev Baiana Saude Publica 32: 159–167 [Portuguese].	48
San Martín JL, Brathwaite O, Zambrano B, Solórzano JO, Bouckenooghe A, et al. (2010) The epidemiology of dengue in the Americas over the last three decades: a worrisome reality. Am J Trop Med Hyg 82: 128–135. doi: http://dx.doi.org/10.4269/ajtmh.2010.09-0346.	9
Santos CH, Sousa FYd, Lima LRd, Stival MM. (2009) Epidemiological profile of dengue in Anapolis municipality, Brazil 2001–2007. Rev Patol Trop 38: 249–259.	50
Siqueira JB, Jr, Martelli CM, Coelho GE, Simplicio AC, Hatch DL. (2005) Dengue and dengue hemorrhagic fever, Brazil, 1981–2002. Emerg Infect Dis; 11: 48–53. doi: http://dx.doi.org/10.3201/eid1101.031091.	38

Siqueira JB, Vinhal LC, Said RFC, Hoffmann JL, Martins J, et al. (2010) Chapter 7. Dengue no Brasil: tendencias e mudancas na epidemiologia, com enfase nas epidemias de 2008 e 2010. In: Saúde Brasil 2010: uma análise da situação de saúde e de evidências selecionadas de impacto de ações de vigilância em saúde. Ministério da Saúde, Secretaria de Vigilância em Saúde, Departamento de Análise de Situação em Saúde. Série G. Estatística e Informação em Saúde. ISBN 978-85-334-1851-6. Brasília: Ministério da Saúde, 372 p. Available: http://portal.saude.gov.br/portal/arquivos/pdf/cap_7_saude_brasil_2010.pdf. Accessed: 27 September 2013.	26
Siqueira Junior JB. (2004) Surveillance of dengue fever in urban area: transmission and spatial analysis [thesis]. Goiânia, Brazil: Universidade Federal de Goiás 1 v. 94 p.	30
Siqueira-Junior JB, Maciel IJ, Barcellos C, Souza WV, Carvalho MS, et al. (2008) Spatial point analysis based on dengue surveys at household level in central Brazil. BMC Public Health 8: 361. doi: http://dx.doi.org/10.1186/1471-2458-8-361.	56
Teixeira Mda G, Costa Mda C, Barreto ML, Mota E. (2005) Dengue and dengue hemorrhagic fever epidemics in Brazil: what research is needed based on trends, surveillance, and control experiences? Cad Saude Publica 21: 1307–15. doi: http://dx.doi.org/10.1590/S0102-311X2005000500002.	29
Teixeira MG, Costa MC, Coelho G, Barreto ML. (2008) Recent shift in age pattern of dengue hemorrhagic fever, Brazil. Emerg Infect Dis 14: 1663. doi: http://dx.doi.org/10.3201/eid1410.071164.	53
Teixeira MG, Costa Mda C, Barreto F, Barreto ML. (2009) Dengue: twenty-five years since reemergence in Brazil. Cad Saude Publica 25(Suppl 1): S7–S18. doi: http://dx.doi.org/10.1590/S0102-311X2009001300002.	15
Teixeira TR, Medronho RA. (2008) [Socio-demographic factors and the dengue fever epidemic in 2002 in the State of Rio de Janeiro, Brazil]. Cad Saude Publica 24: 2160–2170 [Portuguese]. doi: http://dx.doi.org/10.1590/S0102-311X2008000900022.	72
