# Supplementary material for: Epidemiological Trends of Dengue Disease in Brazil (2000–2010): A Systematic Literature Search and Analysis
Source: PLoS Negl Trop Dis. 2013 Dec 19;7(12):e2520. doi: 10.1371/journal.pntd.0002520 (PMC3871634; doi:10.1371/journal.pntd.0002520)
Supplement: Table S2 — Incidence of dengue disease in Brazil: national data. (PDF) [file pntd.0002520.s003.pdf]

Supplementary Table S2. Incidence of dengue disease in Brazil: national data.

Year	DF		Laboratory-confirmed DF (n)	DF hospitalization		DHF (n)	DF-related mortality (n)	Source of data First author, year [ref]
	(n)	Incidence (per 100,000 population)		(n)	Population incidence			
2000	227,957		41,260			62	5	Da Fonseca 2009 [27]
2000	231,000					888	92	Figueiredo 2010 [16]
2000		147				60		Teixeira 2005 [29]
2000		150						Teixeira 2009 [15]
2000		144						Dos Santos 2003 [28]
2000	~230,000					40	3	Nogueira 2002 [6]
2000		92.3			6			Siqueira 2004 [30]
2000	138,388						47/24§	Moraes 2009 [31]
2001	381,718		120,241			682	29	Da Fonseca 2009 [27]
2001	413,000					679	140	Figueiredo 2010 [16]
2001		250				630		Teixeira 2005 [29]
2001		225						Teixeira 2009 [15]
2001		254						Dos Santos 2003 [28]
2001	~410,000					679	29	Nogueira 2002 [6]
2001		225.3		14.1				Siqueira 2004 [30]
2001	406,750						193/60§	Moraes 2009 [31]
2002	794,219		144,548			2714	150	Da Fonseca 2009 [27]
2002	781,000					2714	255	Figueiredo 2010 [16]
2002	691,933	396.2	12,720	55,266	31.6‡	2608	152	Siqueira 2010 [26]
2002		452				2700		Teixeira 2005 [29]
2002		470						Teixeira 2009 [15]
2002		385						Dos Santos 2003 [28]
2002	~700,000							Nogueira 2002 [6]

Year	DF		Laboratory-confirmed DF (n)	DF hospitalization		DHF (n)	DF-related mortality (n)	Source of data First author, year [ref]
	(n)	Incidence (per 100,000 population)		(n)	Population incidence			
2002		335.3			31.6			Siqueira 2004 [30]
2002	684,527						394/199§	Moraes 2009 [31]
2003	280,529		83,878			727	38	Da Fonseca 2009 [27]
2003	342,000					727	164	Figueiredo 2010 [16]
2003	299,019		90,005	54,396		913	89	Siqueira 2010 [26]
2003		195				650		Teixeira 2005 [29]
2003		200						Teixeira 2009 [15]
2003	294,245						232/86§	Moraes 2009 [31]
2004	71,847		26,655			103	8	Da Fonseca 2009 [27]
2004	113,000					81	71	Figueiredo 2010 [16]
2004	77,753		28,924	20,900		159	19	Siqueira 2010 [26]
2004		75						Teixeira 2009 [15]
2004	77,649						68/33§	Moraes 2009 [31]
2005	148,524		53,469			463	45	Da Fonseca 2009 [27]
2005	204,000					1395	159	Figueiredo 2010 [16]
2005	157,286		57,724	32,432		530	78	Siqueira 2010 [26]
2005		150						Teixeira 2009 [15]
2005	134,298						128/59§	Moraes 2009 [31]
2006	252,725		102,354			682	76	Da Fonseca 2009 [27]
2006	347,000					642	193	Figueiredo 2010 [15]
2006	275,833		111,988	32,051		910	147	Siqueira 2010 [26]
2006		200						Teixeira 2009 [15]
2007	507,296		173,495			1586	161	Da Fonseca 2009 [27]
2007	560,000					1541	317	Figueiredo 2010 [16]

Year	DF		Laboratory-confirmed DF (n)	DF hospitalization		DHF (n)	DF-related mortality (n)	Source of data First author, year [ref]
	(n)	Incidence (per 100,000 population)		(n)	Population incidence			
2007	501,666		177,590	53,461		1907	292	Siqueira 2010 [26]
2007		300						Teixeira 2009 [15]
2008	806,036		144,280			4137	229	Da Fonseca 2009 [27]
2008	734,000					647	306	Figueiredo 2010 [16]
2008	637,663	336.3	136,460	77,316	40.8‡	4502	575	Siqueira 2010 [26]
2008		120						Teixeira 2009 [15]
2009	407,000							Figueiredo 2010 [16]
2009	411,500		111,105	54,482		2679	353	Siqueira 2010 [26]
2010	1,027,100	538.4	344,079	94,758	49.7‡	3807	678	Siqueira 2010 [26]

DF, dengue fever; DHF, dengue haemorrhagic fever.

‡Hospitalization rate for DHF.
§Solidus separates results from two different reporting systems: SINAN (first) and SIM (second).
