# Supplementary material for: Epidemiological Trends of Dengue Disease in Brazil (2000–2010): A Systematic Literature Search and Analysis
Source: PLoS Negl Trop Dis. 2013 Dec 19;7(12):e2520. doi: 10.1371/journal.pntd.0002520 (PMC3871634; doi:10.1371/journal.pntd.0002520)
Supplement: Table S3 — Incidence of dengue disease in Brazil: regional data. (PDF) [file pntd.0002520.s004.pdf]

Supplementary Table S3. Incidence of dengue disease in Brazil: regional data.

Year	Location	Region	DF		Laboratory-confirmed DF (n)	DF hospitalization (n)	DHF (n)	DF mortality		Source of data First author, year[Ref]
			(n)	Incidence (per 100,000 population)				(n)	Population incidence	
2000	City of Vitória	Southeast	5679	1942.8	1652		11*	3		Cardoso 2011 [42]
2000	State of Ceará	Northeast	13,645				4[†]			Cavalcanti 2011 [39]
2000	State of Pernambuco	Northeast	27,949				38	-		Cordeiro 2007 [34]
2000	City of Itabuna	Northeast	1279	650.3						De Souza 2010 [40]
2000	State of São Paulo	Southeast		9.74						Gasparetti 2007 [46]
2000	City of Glory of Goitá	Northeast	133	482.68						Dos Santos 2003 [28]
2000	City of Goiânia	Central-West	6709		203					Feres 2006 [45]
2000	City of Ribeirão Preto	Southeast	208	43.45						Hino 2010 [47]
2000	City of São Luís	Northeast	194							Goncalves Neto 2004 [35]
2000	Rio de Janeiro	Southeast	~6000							Nogueira 2002 [6]
2000	City of Salvador	Northeast		1888.5 (Liberdale); 1583.2 (Itapagipe)						Sampaio 2008 [48]
2000	State of Piaui	Northeast	7000							De Castro 2003 [49]
2001	City of Vitória	Southeast	1837	620.6	357		4*	1		Cardoso 2011 [42]
2001	State of Ceará	Northeast	34,930				82[†]			Cavalcanti 2011 [39]

Year	Location	Region	DF		Laboratory-confirmed DF (n)	DF hospitalization (n)	DHF (n)	DF mortality		Source of data First author, year[Ref]
			(n)	Incidence (per 100,000 population)				(n)	Population incidence	
2001	State of Pernambuco	Northeast	17,112				49	1		Cordeiro 2007 [34]
2001	City of Itabuna	Northeast	458	231.5						De Souza 2010 [40]
2001	State of São Paulo	Southeast		137.3						Gasparetti 2007 [46]
2001	States of Paraná, Santa Catarina and Rio Grande do Sul	South (these three states make up all of the South region)	1628				33			Koyama 2009 [43]
2001	City of Goiânia	Central-West	6709		203					Feres 2006 [45]
2001	City of Ribeirão Preto	Southeast	3186	619.65						Hino 2010 [47]
2001	City of São Luís	Northeast	601							Goncalves Neto 2004 [35]
2001	Rio de Janeiro	Southeast	~70,000							Nogueira 2002 [6]
2001	City of Anapolis	Central-West		31						Santos 2009 [50]
2001	State of Piaui	Northeast	10,400							De Castro 2003 [49]
2002	City of Vitória	Southeast	4324	1444.4	4258		11*	0		Cardoso 2011 [42]
2002	State of Ceará	Northeast	16,465				72†			Cavalcanti 2011 [39]
2002	State of Pernambuco	Northeast	116,245				340	20		Cordeiro 2007 [34]
2002	City of Itabuna	Northeast	1868	938.3						De Souza 2010 [40]
2002	State of São Paulo	Southeast		102.62						Gasparetti 2007 [46]

Year	Location	Region	DF		Laboratory-confirmed DF (n)	DF hospitalization (n)	DHF (n)	DF mortality		Source of data First author, year[Ref]
			(n)	Incidence (per 100,000 population)				(n)	Population incidence	
2002	States of Paraná, Santa Catarina and Rio Grande do Sul	South (these three states make up all of the South region)	7318				376			Koyama 2009 [43]
2002	City of Teresina	Northeast	4259				1			Monteiro 2009 [51]
2002	City of Goiânia	Central-West	17,632		697					Feres 2006 [45]
2002	City of Ribeirão Preto	Southeast	347	66.66						Hino 2010 [47]
2002	City of Recife	Northeast	35,597	2502			208	14		Montenegro 2006 [44]
2002	City of São Luís	Northeast	904							Goncalves Neto 2004 [35]
2002	Rio de Janeiro	Southeast	~65,000					40		Nogueira 2002 [6]
2002	City of Salvador	Northeast		1088.9				9	0.35	Sampaio 2008 [48]
2002	City of Anapolis	Central-West	200	61						Santos 2009 [50]
2002	State of Piaui	Northeast	10,300							De Castro 2003 [49]
2003	City of Vitória	Southeast	6358	2100.9	6140		11*	2		Cardoso 2011 [42]
2003	State of Ceará	Northeast	23,796				292†			Cavalcanti 2011 [39]
2003	State of Pernambuco	Northeast	26,083				21	3		Cordeiro 2007 [34]
2003	City of Itabuna	Northeast	5811	2902.8						De Souza 2010 [40]
2003	State of São Paulo	Southeast		52.68						Gasparetti 2007 [46]

Year	Location	Region	DF		Laboratory-confirmed DF (n)	DF hospitalization (n)	DHF (n)	DF mortality		Source of data First author, year[Ref]
			(n)	Incidence (per 100,000 population)				(n)	Population incidence	
2003	States of Paraná, Santa Catarina and Rio Grande do Sul	South (these three states make up all of the South region)	11,050				468			Koyama 2009 [43]
2003	City of Teresina	Northeast	3920				14			Monteiro 2009 [51]
2003	City of Goiânia	Central-West	7438		69					Feres 2006 [45]
2003	City of Ribeirão Preto	Southeast	799	151.4						Hino 2010 [47]
2003	City of Anapolis	Central-West		9						Santos 2009 [50]
2004	City of Vitória	Southeast	502	162.2	324		0*	0		Cardoso 2011 [42]
2004	State of Ceará	Northeast	3094				14[†]			Cavalcanti 2011 [39]
2004	State of Pernambuco	Northeast	6337				16			Cordeiro 2007 [34]
2004	City of Itabuna	Northeast	118	58.62						De Souza 2010 [40]
2004	State of São Paulo	Southeast		7.77						Gasparetti 2007 [46]
2004	States of Paraná, Santa Catarina and Rio Grande do Sul	South (these three states make up all of the South region)	44				58			Koyama 2009 [43]
2004	City of Teresina	Northeast	110				0			Monteiro 2009 [51]
2004	City of Salvador	Northeast		9.52				0.07		Sampaio 2008 [48]
2004	City of Anapolis	Central-West	5	1						Santos 2009 [50]

Year	Location	Region	DF		Laboratory-confirmed DF (n)	DF hospitalization (n)	DHF (n)	DF mortality		Source of data First author, year[Ref]
			(n)	Incidence (per 100,000 population)				(n)	Population incidence	
2005	City of Vitória	Southeast	1235	394.2	228		1*	0		Cardoso 2011 [42]
2005	State of Ceará	Northeast	22,817				199†			Cavalcanti 2011 [39]
2005	State of Pernambuco	Northeast	12,990				21	2		Cordeiro 2007 [34]
2005	City of Itabuna	Northeast	3827	1877.7						De Souza 2010 [40]
2005	State of São Paulo	Southeast		13.58						Gasparetti 2007 [46]
2005	States of Paraná, Santa Catarina and Rio Grande do Sul	South (these three states make up all of the South region)			379		94			Koyama 2009 [43]
2005	City of Teresina	Northeast	153				7			Monteiro 2009 [51]
2005	City of Anapolis	Central-West		10						Santos 2009 [50]
2005	City of Goiânia	Central-West	9531			880/188‡				Da Silva 2009 [41]
2006	City of Vitória	Southeast	2386	752.5	704		3*	1		Cardoso 2011 [42]
2006	State of Ceará	Northeast	25,569				173†			Cavalcanti 2011 [39]
2006	State of Pernambuco	Northeast	18,595				33	4		Cordeiro 2007 [34]
2006	City of Itabuna	Northeast	145	70.7						De Souza 2010 [40]
2006	City of Teresina	Northeast	1372				26			Monteiro 2009 [51]
2006	City of Anapolis	Central-West		20						Santos 2009 [50]

Year	Location	Region	DF		Laboratory-confirmed DF (n)	DF hospitalization (n)	DHF (n)	DF mortality		Source of data First author, year[Ref]
			(n)	Incidence (per 100,000 population)				(n)	Population incidence	
2006	City of Goiânia	Central-West	11,867			1599/1181‡				Da Silva 2009 [41]
2007	City of Vitória	Southeast	966	307.6	218		0*	0		Cardoso 2011 [42]
2007	State of Ceará	Northeast	25,026				280†			Cavalcanti 2011 [39]
2007	City of Itabuna	Northeast	128	62.03						De Souza 2010 [40]
2007	City of Anapolis	Central-West		50						Santos 2009 [50]
2007	City of Goiânia	Central-West	6524			177/586‡				Da Silva 2009 [41]
2008	City of Vitória	Southeast	3529	1110.4	985		26*	2		Cardoso 2011 [42]
2008	State of Ceará	Northeast	44,508				408†			Cavalcanti 2011 [39]
2008	City of Itabuna	Northeast	2459	1158.6						De Souza 2010 [40]
2008	City of Goiânia	Central-West	22,629			450/1441‡				Da Silva 2009 [41]
2009	City of Vitória	Southeast	7657	1554.6	4977		78*	6		Cardoso 2011 [42]
2009	City of Itabuna	Northeast	15,197	7095.6						De Souza 2010 [40]

DF, dengue fever; DHF, dengue haemorrhagic fever; DSS, dengue shock syndrome.

*DHF/DSS/DFC cases.
†Incidence rate.
‡Solidus separates results from two different reporting systems: SINAN (first) and SIM (second).
